# First records of *Canis dirus* and *Smilodon fatalis* from the late Pleistocene Tule Springs local fauna, upper Las Vegas Wash, Nevada

**DOI:** 10.7717/peerj.2151

**Published:** 2016-06-21

**Authors:** Eric Scott, Kathleen B. Springer

**Affiliations:** 1 Division of Geological Sciences, San Bernardino County Museum, Redlands, CA, United States of America; 2 Current affiliation: Dr. John D. Cooper Archaeological and Paleontological Center, California State University, Fullerton, CA, United States of America; 3 Current affiliation: Geosciences and Environmental Change Science Center, United States Geological Survey, Denver, CO, United States of America

**Keywords:** Tule Springs, *Canis dirus*, *Smilodon fatalis*, Rancholabrean, Las Vegas Wash

## Abstract

Late Pleistocene groundwater discharge deposits (paleowetlands) in the upper Las Vegas Wash north of Las Vegas, Nevada, have yielded an abundant and diverse vertebrate fossil assemblage, the Tule Springs local fauna (TSLF). The TSLF is the largest open-site vertebrate fossil assemblage dating to the Rancholabrean North American Land Mammal Age in the southern Great Basin and Mojave Desert. Over 600 discrete body fossil localities have been recorded from the wash, including an area that now encompasses Tule Springs Fossil Beds National Monument (TUSK). Paleowetland sediments exposed in TUSK named the Las Vegas Formation span the last 250 ka, with fossiliferous sediments spanning ∼100–13 ka. The recovered fauna is dominated by remains of *Camelops*and *Mammuthus*, and also includes relatively common remains of extinct *Equus*and *Bison*as well as abundant vertebrate microfaunal fossils. Large carnivorans are rare, with only *Puma concolor* and *Panthera atrox* documented previously. Postcranial remains assigned to the species *Canis dirus* (dire wolf) and *Smilodon fatalis* (sabre-toothed cat) represent the first confirmed records of these species from the TSLF, as well as the first documentation of *Canis dirus* in Nevada and the only known occurrence of *Smilodon*in southern Nevada. The size of the recovered canid fossil precludes assignment to other Pleistocene species of *Canis*. The morphology of the felid elements differentiates them from other large predators such as *Panthera*, *Homotherium*, and *Xenosmilus*, and the size of the fossils prevents assignment to other species of *Smilodon*. The confirmed presence of *S. fatalis* in the TSLF is of particular interest, indicating that this species inhabited open habitats. In turn, this suggests that the presumed preference of *S. fatalis* for closed-habitat environments hunting requires further elucidation.

## Introduction

The iconic Pleistocene mammalian predators *Canis dirus* Leidy, 1858 and *Smilodon fatalis* Leidy, 1868 are frequent components of many late Pleistocene vertebrate faunas throughout midcontinental North America (Kurtén & Anderson, 1980). At many of these localities, however, both species are represented by limited skeletal material. In contrast, literally thousands of individual animals of each species are represented in the assemblage from the Rancho La Brea “tar pits” in Los Angeles, California (Stock, 1992).

In the southern Great Basin and Mojave Desert, Pleistocene vertebrate assemblages are common and include open sites, caves, and middens (Jefferson, McDonald & Livingston, 2004; Scott & Cox, 2008). However, remains of *Canis dirus* are uncommon, known only from the Bitter Spring playa locality on Fort Irwin, and from an additional record of *Canis* sp. cf. *C. dirus* from Lake Manix, both in the west-central Mojave in California (Reynolds & Reynolds, 1994; Scott, 2000). Similarly, fossils of *Smilodon* are poorly represented from the region; prior to the present study, *Smilodon* sp. cf. *S. fatalis* has been reported only from Twentynine Palms (Bacheller III, 1978), whereas *Smilodon* sp. was reported from China Lake (Fortsch, 1978) and Fort Irwin (Reynolds & Reynolds, 1994; Scott, 2000).

Both *Canis dirus* and *Smilodon fatalis* are conspicuously absent from published records describing fossils recovered from the Tule Springs locality and surrounding upper Las Vegas Wash north of Las Vegas, Nevada (Simpson, 1933; Mawby, 1967). In this region, extensive outcrops of groundwater discharge deposits (paleowetlands) are highly fossiliferous (Simpson, 1933; Haynes, 1967; Mawby, 1967; Quade, 1986; Quade & Pratt, 1989; Reynolds, Mead & Reynolds, 1991; Scott & Cox, 2008), and faunal remains have been designated recently as the Tule Springs local fauna (TSLF) (Springer et al., 2011). The TSLF is the largest open-site late Pleistocene vertebrate assemblage known from the southern Great Basin and Mojave Desert. This report documents the first records of *C. dirus* and *S. fatalis* in the TSLF and the first definitive records of these taxa in southern Nevada.

## Geologic Setting and Age Control

**Figure 1:**
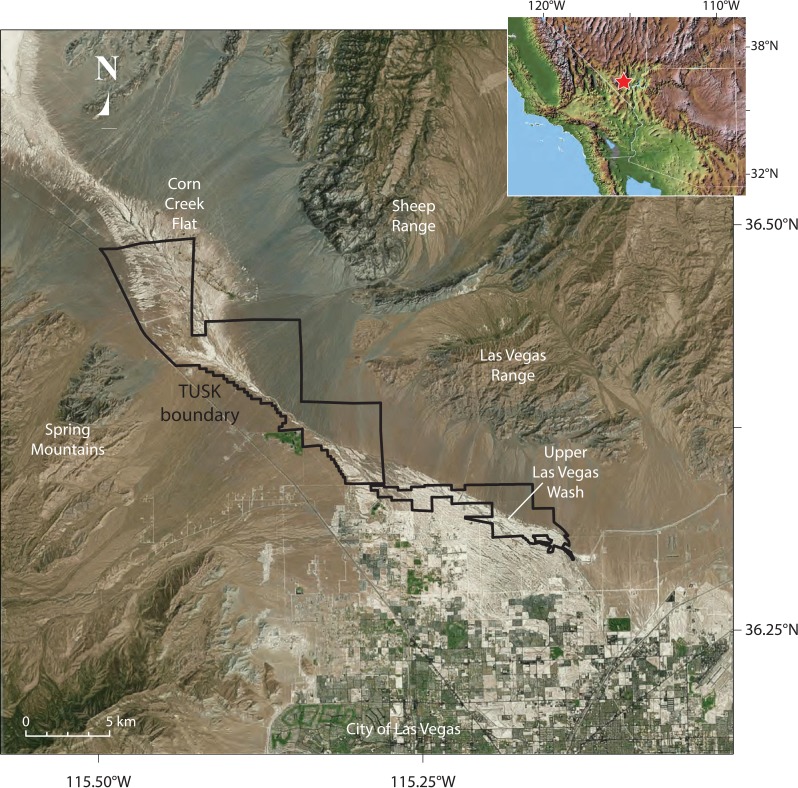
Aerial view, location of Tule Springs Fossil Beds National Monument (TUSK). The borders of the monument and key geographic features are noted.

During the late Pleistocene and early Holocene, the Las Vegas Valley in southern Nevada (Fig. 1) supported extensive and diverse wetland ecosystems. Desert wetlands form in arid environments where water tables approach or breach the ground surface and are expressed on the landscape by a variety of hydrologic settings, including seeps, marshes, wet meadows, spring pools, and flowing streams. Over time, eolian and alluvial sediments become trapped by dense vegetation and wet ground conditions, resulting in a unique combination of clastic sediments, chemical precipitates, and organic matter that are collectively referred to as groundwater discharge (GWD) deposits (Pigati et al., 2014). During the late Pleistocene, high-water-table elevations supported vast wetlands that were distributed along the length of the Las Vegas Valley, which in turn supported a diverse flora and fauna (Springer, Manker & Pigati, 2015). The resulting GWD deposits once blanketed much of the Las Vegas Valley floor and areas to the north and were assigned to the Las Vegas Formation (Longwell et al., 1965), although they were initially misinterpreted as a pluvial lake sequence. As a result of urbanization, these once extensive deposits are presently restricted to the upper Las Vegas Wash, the major physiographic drainage of the Las Vegas region and a tributary of the Colorado River (Fig. 1). Here, GWD deposits occur as light-colored, fine-grained sedimentary sequences with distinctive badland topography (Longwell et al., 1965; Haynes, 1967; Quade, 1986; Quade et al., 1995; Quade, Forester & Whelan, 2003; Ramelli et al., 2011; Ramelli et al., 2012; Springer, Manker & Pigati, 2015).

Haynes (1967) informally subdivided the Las Vegas Formation into stratigraphically ascending units designated A through G, of which A–E date to the late Pleistocene and F and G represent the Holocene portion of the sequence. Recent studies (Ramelli et al., 2011; Ramelli et al., 2012; Springer, Manker & Pigati, 2015) have augmented, revised, and expanded this geochronologic and lithostratigraphic framework, while at the same time retaining the alpha notation for the members of the formation. The new frameworks were established using detailed geologic mapping, stratigraphic principles, and radiocarbon (^14^C) dating of charred vascular plants and, to a lesser extent, small terrestrial gastropod shells, as well as luminescence dating (Ramelli et al., 2011, Ramelli et al., 2012; Springer, Manker & Pigati, 2015).

The specimen assigned herein to *Canis dirus* was recovered from locality SBCM 2.6.641, in sediments identified as Bed E_1*b*_ of the Las Vegas Formation (Springer, Manker & Pigati, 2015). This bed represents a discrete discharge event dating to between 14.6 and 14.3 ka, which places this specimen of *C. dirus* temporally near the end of the Pleistocene.

Fossils referred herein to *Smilodon fatalis* derive from locality SBCM 2.6.136, in sediments identified as Bed E_1*a*_ of the Las Vegas Formation, representing an older, discrete discharge event. Bed E_1*a*_ dates to 16.1–15.0 ka cal BP (Springer, Manker & Pigati, 2015), thereby also establishing the presence of *S. fatalis* in the southern Nevada region towards the end of the Pleistocene Epoch.

## Paleoenvironment

The depositional environments represented by the GWD deposits in the Las Vegas Valley are both spatially complex and time-transgressive, with disparate sedimentological facies related to hydrologic discharge regime and topographic position in the valley. The former desert wetlands, with their attendant vertebrate faunas, are analogous to modern spring ecosystems exhibiting helocrene (wet meadows and marshes), rheocrene (flowing streams), and limnocrene (spring-fed pools) discharge regimes (Springer, & Stevens, 2009; Springer, Manker & Pigati, 2015). Chronologic and lithostratigraphic data suggest that there were multiple outflow streams (rheocrene discharge) along the upper Las Vegas Wash during the late Pleistocene. Specimens of both *Canis dirus* and *Smilodon fatalis* were recovered from these groundwater discharge fed outflow streams, and the deposits yielding *C. dirus* are inset into those yielding *S. fatalis*.

The discharge event represented by Bed E_1*a*_ occurred between 16.1 ka and 15.0 ka, corresponding to the Oldest Dryas global cooling event, also termed the “Big Wet” in the southwestern US (Broecker et al., 2009). Abundant vertebrate fossils of multiple taxa, including the fossils of *Smilodon* described below, occur within this unit. Groundwater discharge ended abruptly at 15.0 ka, as evidenced by intense erosion of the deposits in response to the initiation of the Bølling warm period (D-O 1), but resumed shortly thereafter as represented by Bed E_1*b*_. This later discharge interval, again dating to 14.6 and 14.3 ka, is also prolific with respect to vertebrate fossils, including the specimen of *Canis dirus* reported herein.

Macrofloral evidence obtained from charred organic material in the discharge interval from which the fossil of *Canis dirus* was obtained indicates mesquite, conifer and possibly juniper were present in the Las Vegas Valley during this period (Springer, Manker & Pigati, 2015). Mehringer’s (1965, 1967) pollen data suggested a lowering of vegetation zones by nearly 1,000 m, corroborating macrofloral evidence of conifers at the valley bottom of the Las Vegas Valley at an elevation of 700 m above sea level. Other plant associations, based on pollen and woodrat midden studies, suggest that an open sagebrush-dominated steppe to lower Mojave Desert scrub prevailed in the valley during the late Pleistocene (Quade, 1986).

Mehringer (1967) advocated that during the late Pleistocene, conditions were much like those observed today on the slopes of the mountain ranges ringing the Las Vegas Valley, a sagebrush desert bordered by piñon-juniper woodland. The long-term desertification of this area (on glacial to interglacial timescales) was in progress during the time, and was punctuated by discharge events, in which the sediments yielding both *Canis dirus* and *Smilodon fatalis* were deposited.

## Methods

This study was conducted from 2008 to 2014 on ∼11,000 acres of federal land managed by the Bureau of Land Management (BLM), Southern Nevada District Office. Work was performed under Grant L08AC13098 entitled “The Upper Las Vegas Wash Conservation Transfer Area, Clark County, NV: Treatment, Protection, and Interpretation of Heritage Paleontological Resources through Public Involvement.”

Specimens assigned herein to *Canis dirus* and *Smilodon fatalis* were examined and measured in the collections of the San Bernardino County Museum; comparative fossils were studied and measured at the La Brea Tar Pits and Museum (formerly the George C. Page Museum of La Brea Discoveries). Measurements followed procedures outlined by Von den Driesch (1976) as appropriate and where possible; however, the poor preservation of the fossils necessitated developing additional measurements in order to better constrain the size of some specimens. Data were acquired using Mitutoyo Digimatic calipers connected by a Mitutoyo USB digital interface to a Toshiba Satellite P745 laptop. Comparative metric data for living *Canis lupus* from Yellowstone National Park and Alaska were provided by Sue Ware. Digital photos were acquired with a Sony DSC F818 Cybershot camera. Graphic plots were generated and basic statistics calculated using SigmaPlot 13. Multiple logistic regression was performed using online software provided by StatPages.org (http://statpages.org/logistic.html).

## Results

### Systematic paleontology

**Table:** 

Order Carnivora Bowdich, 1821
Family Canidae Fischer von Waldheim, 1817
Genus *Canis* Linnaeus, 1758
*Canis dirus* Leidy, 1858

**Referred specimens:** SBCM L3160-1257, right patella.

**Figure 2:**
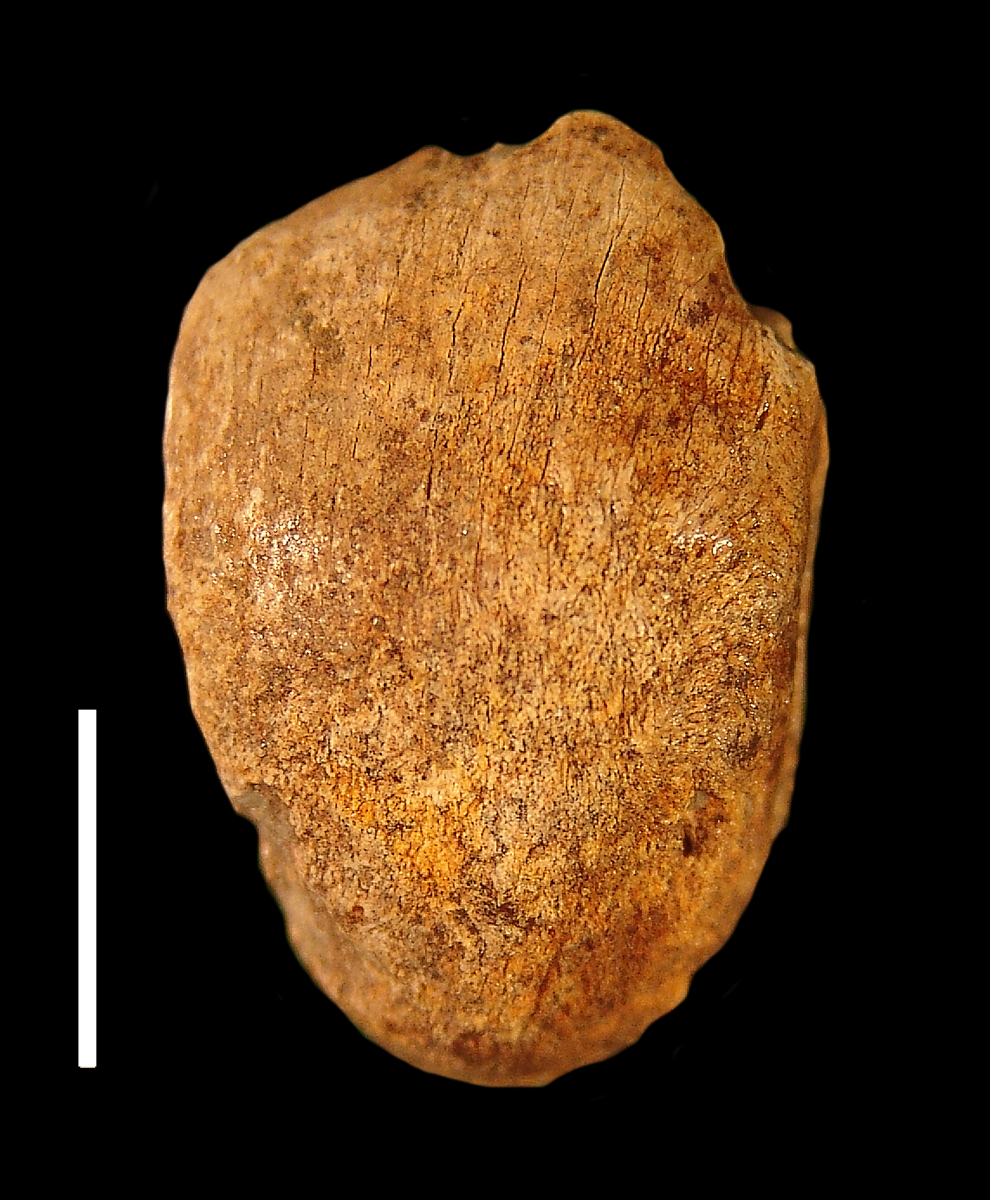
SBCM L3160-1257, right patella of *Canis dirus*, dorsal view. Scale = 1 cm.

**Description:** The patella is damaged dorsally, but nevertheless exhibits a typically canid rounded teardrop shape (Fig. 2).

**Figure 3:**
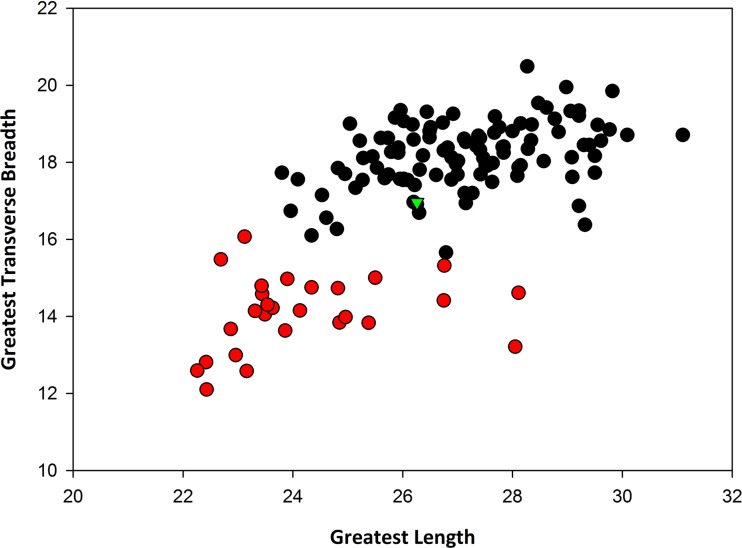
Bivariate plot of greatest length vs. transverse breadth, in millimeters, of patellae of *Canis dirus* from Rancho La Brea (black circles), *C. lupus* from Yellowstone and Alaska (red circles), and SBCM L3160-1257 from TUSK (green triangle).

**Remarks:** In terms of size, SBCM L3160-1257 is far too large to be assigned to *Canis latrans*, and additionally is larger than like elements of timber wolf, *C. lupus* (Table 1). Metric comparisons with fossils of *C. dirus* from Rancho La Brea, as well as with modern specimens of *C. lupus* from Yellowstone National Park and from Alaska, demonstrate that L3160-1257 falls well within the size range of *C. dirus* (Fig. 3). Applying multiple logistic regression confirms this interpretation (*P* = 1.0, *df* = 2, 99% CI).

**Table 1:** Metric data from patellae of *Canis dirus* (Rancho La Brea) and modern *C. lupus* from Yellowstone National Park and Alaska, compared with measurements of SBCM L3160-1257 from TUSK.

Taxon:	*Canis dirus* from RLB		*Canis lupus* (modern)		*Canis dirus* from TUSK	
Data:	L	W	L	W	L	W
N	107	107	29	29	1	1
Max	31.1	20.49	28.11	16.07	26.26	16.96
Min	23.8	15.66	22.26	12.10		
Mean	27.13	18.19	24.23	14.10		
Median	27.06	18.25	23.63	14.15		
SD	1.55	0.86	1.63	0.95		

**Notes.**

L Length W Transverse width

Data in millimeters.

SBCM L3160-1257 was recovered from locality SBCM 2.6.641, which also yielded fragmentary but identifiable fossil remains of *Lepus*, cf. *Mammuthus*, *Camelops*, and *Odocoileus*.

**Table:** 

Family Felidae Gray, 1821
Subfamily Machairodontinae Gill, 1872
Genus *Smilodon* Lund, 1841
*Smilodon fatalis* Leidy, 1868

**Referred specimens:** SBCM L3160-1018, proximal left humerus; SBCM L3160-1019, distal left radius; SBCM L3160-1020, dorsal sacrum.

**Description:** All of the recovered fossils are poorly preserved. Observed damage appears to have been post-depositional and post-exposure. Breakage is confined to fractures running roughly parallel with, as well as perpendicular to, the long axis of the bones; no spiral fractures, indicative of fresh or “green” bone breakage, are in evidence. The compact bone shows no indications of exfoliation, desiccation, abrasion, weathering, or water wear, indicating that the fossils were not transported any great distance and were not exposed to the elements for very long prior to burial. Rather, damage and loss of missing portions of the fossils is inferred to result from diagenetic processes and prolonged exposure to the elements subsequent to erosion but prior to discovery.

**Figure 4:**
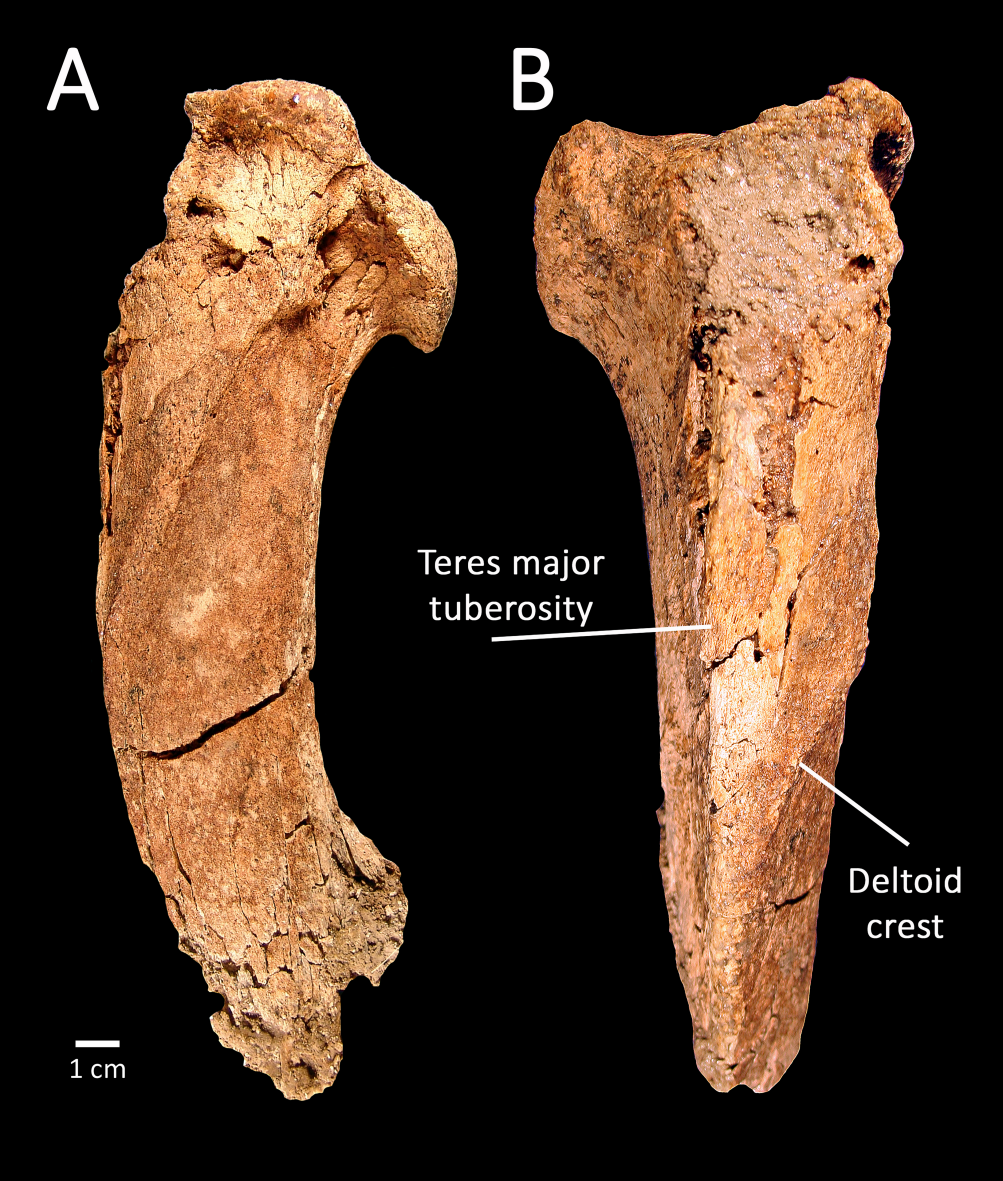
SBCM L3160-1018, proximal left humerus of *Smilodon fatalis*, lateral (A) and anterior (B) views. Key anatomical landmarks are noted.

The proximal left humerus, SBCM L3160-1018, is the most complete of the preserved elements, and falls within the size range of both *Smilodon fatalis* and *Panthera atrox* (Table 2). The specimen lacks the greater tuberosity but is otherwise largely complete proximal to midshaft (Fig. 4). Viewed dorsally, the diaphysis is broad and massive, while in lateral view it exhibits a gentle dorsoventral curvature. Viewed anteriorly, the teres major tuberosity of SBCM L3160-1018 is relatively straight, paralleling the long axis of the diaphysis, while the deltoid crest forms the outer border of the bone proximally, then angles sharply medially to intersect the teres major tuberosity proximal to midshaft.

**Table 2:** Metric data from humeri of *Smilodon fatalis* and *Panthera atrox* from Rancho La Brea (RLB), compared with measurements of SBCM L3160-1018 from TUSK. “Bp,” proximal breadth, “DVH,” minimum dorsoventral depth of head of humerus, “LT,” length of lesser tubercle. The latter two measurements were specifically designed for this study in order to successfully retrieve useful metric data from SBCM L3160-1018, due to the damaged nature of the fossil. All data in millimeters.

Taxon:	*Smilodon fatalis* from RLB			*Panthera atrox* from RLB			*Smilodon fatalis* from TUSK		
**Data:**	Bp	DVH	LT	Bp	DVH	LT	Bp	DVH	LT
N	101	101	101	24	24	24	1	1	1
Max	95.68	75.84	66.38	102.74	82.23	61.16	85.21	76.38	49.63
Min	68.34	57.75	39.74	75.13	63.24	46.20			
Mean	82.81	66.79	48.88	88.20	72.41	52.74			
Median	82.35	66.78	48.47	88.15	71.68	52.12			
SD	5.30	3.51	3.77	6.08	4.17	3.51			

**Figure 5:**
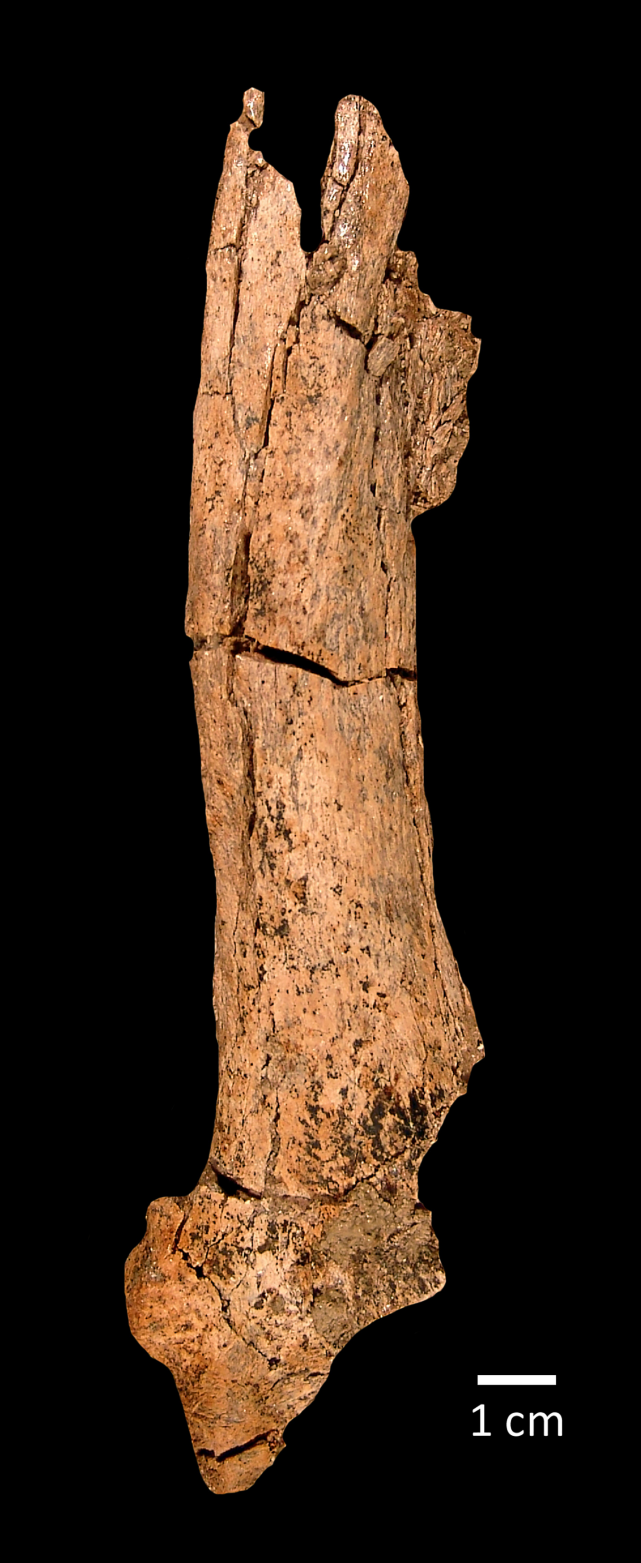
SBCM L3160-1019, distal left radius of *Smilodon fatalis*, dorsal view.

The distal left radius, SBCM L3160-1019, lacks the proximal half and much of the medial face (Fig. 5). The specimen is insufficiently complete to permit obtaining any useful metric data. The distal diaphysis is clearly bowed. The medial border of the styloid process is moderately long and curved or bulbous. The lateral border of the radius curves inward directly from the ulnar articulation. The muscle scar for the insertion of the *m. pronator teres* is situated well distal to midshaft on the diaphysis.

The partial sacrum, SBCM L3160-1020, lacks most of the ventral portion, including the centra. The remaining dorsal surface does preserve some diagnostic features, however. In particular, the prezygapophyses are relatively small and ovular, and are flanked laterally by a distinct and deep groove that extends from behind each process anteriorly, running between the zygapophyses and the alae.

**Remarks:** The morphology of the teres major tuberosity of SBCM L3160-1018 confirms that the humerus is machairodont; in other large felines such as *Panthera atrox*, the teres major tuberosity is angular rather than straight, curving laterally from roughly midline to meet the medially angling deltoid crest. The diaphysis of the humerus SBCM L3160-1018 is more robust and less elongate in dorsal view than the observed condition in humeri of *Homotherium*, precluding assignment of this specimen to that genus. In like manner, the anteroposterior curvature of the diaphysis, best seen in lateral view, matches the curvature seen in *Smilodon fatalis* rather than the straighter profile characterizing humeri of *Xenosmilus*.

The distal diaphysis of radius SBCM L3160-1019 is clearly bowed as in *Smilodon*, rather than straight as exhibited by *Panthera atrox*. The medial border of the styloid process is moderately long and curved or bulbous, closely in accord with radii of *Smilodon* and differing from the longer, straighter processes exhibited by *P. atrox* as well as the smaller, more rounded processes seen in *Homotherium* and *Xenosmilus*. The lateral border of the radius curves inward directly from the ulnar articulation, as seen in *Smilodon* but differing from the longer, straighter condition characterizing *P. atrox*. The muscle scar for the insertion of the *m. pronator teres* is more distally situated on the diaphysis, again as in *Smilodon* as opposed to *P. atrox*.

The prezygapophyses exhibited by SBCM L3160-1020 are relatively small and ovular, and are flanked laterally by a distinct and deep groove that extends from behind each process anteriorly, running between the zygapophyses and the alae. These features are characteristic of *Smilodon*, but differ from the anatomical configuration exhibited by *Panthera atrox* (Merriam & Stock, 1932).

All of the recovered specimens derive from a single locality, SBCM 2.6.136. The locality was initially discovered and recorded in September of 2003, with the two partial limb elements exposed. Original excavation of the fossils took place in June of 2012; upon recognition that the elements preserved represented *Smilodon*, a second field visit to expand the quarry yielded the partial sacrum. No other taxa were recovered from this locality.

## Discussion

Prior to the present study, the only large (≥44 kg) predator known definitively from the Las Vegas Formation was the extinct North American lion *Panthera atrox* (Mawby, 1967). Fossil remains of this extinct species were rare, even though a phalanx of *P. atrox* was among the first fossils discovered in the valley and preserved by paleontologists in 1919 (Springer et al., 2011). The paucity of fossils of large carnivorans was consistent with the interpretation that the fossils from Tule Springs represented a “normal” distribution, wherein herbivores are far more numerous than predators (e.g., Schaller, 1972). In this context, the absence of remains of other large predators such as dire wolf *Canis dirus* and the sabre-toothed cat *Smilodon fatalis* was not considered unusual.

The new records of *Canis dirus* and *Smilodon fatalis* from the TSLF add significantly to the sparse record of late Pleistocene carnivorans from Nevada. *C. dirus* has not previously been confirmed in the state, while the only records of *S. fatalis*in Nevada are from Smith Creek cave (Kurtén & Anderson, 1980) and the Black Rock Desert (Dansie, Davis & Stafford Jr, 1988; Livingston, 1991), both late Pleistocene sites located north of the Mojave Desert.

A possible record of dire wolf was recently reported in the upper Las Vegas Wash from land belonging to the State of Nevada (Rowland & Bonde, 2015). Based on photographic evidence, the fossil in question, a distal metapodial, appears larger than metapodials of coyote (*C. latrans*) and domestic dog (*C. familiaris*). However, metric data discriminating the fossil from other Pleistocene canines such as timber wolf (*C. lupus*) have not been published, and although the specimen was determined to be “indistinguishable from *C. dirus* medial metapodials … it is also indistinguishable from gray wolf (*C. lupus*) medial metapodials” (Rowland & Bonde, 2015: 305–306). Despite this lack of diagnosticity, the fossil was interpreted to be “probably” dire wolf (Rowland & Bonde, 2015: 306), e.g., *Canis* sp. cf. *C. dirus*, based on the relative abundance of *C. dirus* in coastal southern California.

The specimen in question was reported from “Unit D” of the Las Vegas Formation, using the stratigraphy of Haynes (1967) rather than more recent stratigraphic interpretations (e.g., Ramelli et al., 2011; Springer, Manker & Pigati, 2015). Thus, the age of the fossil was estimated to be between 30 and 16 ^14^C ka (∼34–19 cal ka), with no other chronometric age control (Rowland & Bonde, 2015: 306). If confirmed, this fossil would substantially expand the temporal range of *C. dirus* in the Las Vegas Valley, as well as in the State of Nevada. However, lacking any published metric data, we prefer to recognize this fossil as *Canis* sp. (large), instead of *Canis* sp. cf. *C. dirus* until the necessary corroborative metric data are made available.

In contrast, the depositional history, paleoenvironmental setting, and chronology of the deposits yielding the new records of *Smilodon fatalis* and *Canis dirus* are well documented. The lithostratigraphy indicates significant rheocrene discharge occurred at multiple point sources along the upper Las Vegas Wash during the late Pleistocene (Springer, Manker & Pigati, 2015). Given the ongoing desertification of the Las Vegas Valley and the presence of sagebrush desert bordered by piñon-juniper woodland at this time (Mehringer, 1967), these discharge points and their outflow streams would have offered localized sources of fresh water for the regional fauna in an otherwise arid ecosystem. Megafaunal herbivores attracted to this discharge would in turn have offered focused points of interest for carnivorans such as *Canis* and *Smilodon*.

Meloro et al. (2013) examined environmental preferences of living felids and employed humeral morphology to infer paleohabitats for extinct felids, including machairodontines, and concluded that the species *Smilodon populator* was best interpreted to have been adapted to closed habitats. In contrast, the presence of *S. fatalis* documented herein from the upper Las Vegas Wash demonstrates that some species of *Smilodon* also thrived in relatively open habitats, as inferred from the available geological evidence. Published palynological data indicate that the Tule Springs region was an open sagebrush desert with surrounding piñon-juniper woodland during the late Pleistocene (Mehringer, 1967). Both the depositional setting and sedimentology of the GWD deposits yielding the remains of *S. fatalis* indicate spring-derived, low-energy outflow streams, as they are composed predominantly of fine-grained sand and silt (Springer, Manker & Pigati, 2015). The specimens were recovered very near the point source of the discharge (<1 km), demonstrating they did not undergo significant postmortem transport. This is supported by the lack of any evidence of abrasion, rounding, or other water wear of the bones; as noted previously, all observed breakage is post-depositional and post-exposure. This being the case, the fossils are interpreted to have been directly deposited in the open-habitat Tule Springs environment, rather than transported from other, more closed habitats elsewhere. The presence of *S. fatalis* at Tule Springs therefore negates any inferred similarity in closed-habitat preferences between it and *S. populator*.

The presence of *Smilodon fatalis* in open-habitat environments does not preclude considering these animals to have been ambush predators. In fact, multiple studies of the anatomical configuration of *Smilodon* argue convincingly in favor of these felids having been better adapted to ambush rather than pursuit (e.g., Gonyea, 1976; Anyonge, 1996; Meachen-Samuels & Van Valkenburgh, 2010). The new record of *S. fatalis* from the TSLF demonstrates that these animals were not restricted to closed habitats, irrespective of their chosen method of predation. Assumptions that *Smilodon* would have required closed habitats in order to hunt effectively as ambush predators require reconsideration.

Meachen, O’Keefe & Sadleir (2014) proposed that *Smilodon fatalis* evolved morphologically throughout much of the latter part of the Pleistocene, based upon the rich record of the species preserved at Rancho La Brea. Specifically, mandibles of *S. fatalis* exhibited larger average size and a more derived morphology, presumably in response to changing climate, in Pleistocene accumulations dating to 14–13 cal ka (Meachen, O’Keefe & Sadleir, 2014). The fossils of *S. fatalis* from the TSLF did not include mandibular remains, and so direct comparisons with the Rancho La Brea mandibles are not possible. However, measurements from the humerus SBCM L3160-1018 from TUSK are larger than the mean and median measurements obtained from humeri of *Smilodon* at Rancho La Brea (Table 2), and in one dimension—dorsoventral depth of the humeral head (DVH)—exceed the measured sample from the California locality. The large size of the fossils from TUSK is therefore not inconsistent with the interpretation that *S. fatalis* increased in size towards the end of the Pleistocene (Meachen, O’Keefe & Sadleir, 2014). The TUSK fossils are between 16.1 ka and 15.0 ka, and so are older than the youngest Rancho La Brea samples.

## Conclusions

The present study documents the first confirmed records of *Canis dirus* and *Smilodon fatalis* from the Tule Springs site in the eastern Mojave Desert region of Nevada. These finds represent the first documentation of *C. dirus* in Nevada and the only known occurrence of *Smilodon* in southern Nevada. The presence of *S. fatalis* in the assemblage indicates that this species inhabited open habitats. Suggestions that *Smilodon* would have preferred or required closed habitats in order to function successfully as an ambush predator require further consideration.

##  Supplemental Information

10.7717/peerj.2151/supp-1 Supplemental Information 1 Metric data for patellae of C. lupus, GCPM; collected by E Scott

10.7717/peerj.2151/supp-2 Supplemental Information 2 Raw data, humeri of Panthera atrox from Rancho La Brea, CA; data collected by E Scott

10.7717/peerj.2151/supp-3 Supplemental Information 3 Raw data, humeri of Smilodon fatalis from Rancho La Brea, CA; data collected by E Scott

10.7717/peerj.2151/supp-4 Supplemental Information 4 Raw data, humerus of Smilodon fatalis from TUSK; data collected by E Scott

10.7717/peerj.2151/supp-5 Supplemental Information 5 Metric data for patellae of C. dirus from GCPM; collected by E Scott

10.7717/peerj.2151/supp-6 Supplemental Information 6 Metric data for patella of C. dirus from TUSK; data collected by E Scott

## Additional Information and Declarations

### Field Study Permissions

The following information was supplied relating to field study approvals (i.e., approving body and any reference numbers): Bureau of Land Management (BLM), Southern Nevada District Office. Work was performed under Grant L08AC13098 entitled “The Upper Las Vegas Wash Conservation Transfer Area, Clark County, NV: Treatment, Protection, and Interpretation of Heritage Paleontological Resources through Public Involvement.”

### Data Availability

The following information was supplied regarding data availability: The raw data has been supplied as a Supplemental Information.
